# Evaluation of Evidence-Based Practice Competency among Greek Undergraduate Nursing Students

**DOI:** 10.3390/healthcare12181811

**Published:** 2024-09-10

**Authors:** Eleni Miliara, Athena Kalokairinou, Stefania Schetaki, Evridiki Patelarou, Athina Patelarou

**Affiliations:** 1 Department of Nursing, Faculty of Health Sciences, Hellenic Mediterranean University, 71410 Heraklion, Greece; ddk33@edu.hmu.gr (S.S.); epatelarou@hmu.gr (E.P.); apatelarou@hmu.gr (A.P.); 2 Department of Nursing, Faculty of Health Sciences, National and Kapodistrian University of Athens, 11527 Athens, Greece; athkal@nurs.uoa.gr

**Keywords:** evidence-based practice, nursing, undergraduate nursing students, competency

## Abstract

For several years, the global scientific community has accepted and recognized the importance of evidence-based practice for nursing science. The main factor for the implementation of evidence-based practice is the competence of undergraduate nursing students towards evidence-based practice, so that they as active nurses are ready for its application in their clinical practice, in order to provide better care for their patients. The aim of the present study is to examine the level of evidence-based practice competency evaluated with the self-reported Evidence-Based Practice Competence Questionnaire. It is important to mention that such a study has not been conducted on nursing students in Greece, but only on registered nurses. A quantitative study based on cross-sectional design was conducted from February to June 2022 in a convenience sample of Greek undergraduate nursing students. The SPSS 26.0 program was used to perform descriptive, bivariate, and multivariate analyses. A total of 175 undergraduate students participated at the Hellenic Mediterranean University Department of Nursing (Greece), specifically from the 2nd and 3rd academic years. The mean score of the Evidence-Based Practice Competence Questionnaire for nursing undergraduate students was 3.03 ± 0.26, indicating moderate evidence-based practice. Among the Evidence-Based Practice Competence Questionnaire dimensions, mean scores of 3.03 ± 0.32 for attitude toward evidence-based practice, 3.01 ± 0.49 for skills in evidence-based practice, and 3.03 ± 0.49 for knowledge in evidence-based practice were obtained. Significant differences among participants’ mean Evidence-Based Practice Competence Questionnaire scores regarding gender (*p* = 0.766), age (*p* = 0.400), academic year (*p* = 0.153), and training in the field of research methodology (*p* = 0.538) were not found. It appears that the level of readiness towards evidence-based practice is mediocre among undergraduate nursing students at a university in Greece. Therefore, it is necessary to carry out new studies in the future, so that there is a correct approach of all the elements that contribute to the readiness of nursing undergraduate students regarding evidence-based practice.

## 1. Introduction

Evidence-based practice (EBP) is now characterized as essential in order to ensure health benefits in patients. Increasing research is focusing on the subject and implementation of EBP by nurses, who are the most important part found in the health system [1,2]. There was already talk about EBP even before the 1970s. Since 1970, its recognition has been obvious and fully accepted. EBP is a great means of improving nursing care, which serves in better health spending by enhancing the quality of provided health services by increasing the professional satisfaction of nurses [3,4,5].

One important quality in a nurse who provides high-quality care safely and responsibly is the competence in EBP [2,5,6]. Nonetheless, studies show that it is lacking in higher education and, particularly, in the undergraduate level of teaching about EBP, which is a problem for the undergraduate student’s competence on the subject of EBP. The lack of competence concerning EBP makes it difficult for a large number of nurses because they are unable to assess and critically evaluate a piece of information as there was no undergraduate guidance on the subject of EBP [3,6,7,8].

The educational training of undergraduate nursing students is not easy to carry out regarding the adoption and implementation of EBP [9,10,11]. The complexity that is prevalent is great, although many would find it an easy puzzle to solve. The evolution of nursing curricula is essential not only in the teaching of the implementation and adoption of EBP, but also in all other courses, as the development of studies and the generation of new data are increasingly changing [2,5,12,13]. The planning of an EBP program must be performed with deferential care and focus on undergraduate students’ skills and the teaching concept in general. It is believed that the proper teaching method of EBP to educational institutions’ undergraduate nursing students is a focal point for the competence of these students in the field of EBP and must be able to fully develop their critical thinking and perception [9,12,14,15]. In order to achieve the proper guidance and education of undergraduate nursing students in the field of EBP, there needs to be the appropriate infrastructure, technological equipment, and all the knowledge material required to integrate an EBP course into the curriculum and also to assist other courses related to EBP. If this is accomplished then it will be possible to study the degree of effectiveness of these courses, i.e., whether or not the level of knowledge and skills of undergraduate nursing students regarding EBP has improved [2,5,9].

Curricula always have room for development, even more so when it comes to courses related to EBP [16]. However, ambiguity is identified in the efficiency of teaching methods regarding the adoption of EBP by undergraduate students. Although some development in implementation is recognized, their level of knowledge and competence towards EBP remains unstable [3,7]. Nurses seem to recognize their shortcomings in the level of knowledge about EBP and thus refrain from, due to difficulty, evaluating and criticizing research material [7,17,18]. That is why there is constant vigilance by researchers regarding the training and competence of nurses in EBP strategies from the beginning of the undergraduate level of their studies [7,19]. As mentioned above, most active nurses have not received a university education regarding EBP, before its model was fully accepted, so this is another negative aspect of nurses’ competence regarding EBP [5,17]. It is auspicious, however, that there is a reference to the possibility of improving the relationship between the academic and clinical fields of nursing regarding the competence of undergraduate students in EBP for subsequent correct nursing practices [6,9,17,19]. Therefore, the primary pursuit of nursing must remain the thorough education of undergraduate students for the benefit of the patient, in other words, providing the highest quality in the provision of healthcare, which in turn results in a well-structured health system [2,20,21,22].

The present study aims to examine the level of evidence-based practice competence which is evaluated by the Evidence-Based Practice Competence Questionnaire, and therefore is considered important because it provides a better opinion about the competence of undergraduate nursing students regarding EBP so that their nursing ability can be improved in the future. Our research hypothesis on the mean EBP competence level among nursing students is that it is moderate to low and that specific factors including knowledge, skills, and attitudes towards EBP and specific sample characteristics (e.g., age, gender, year of study, etc.) may affect the overall EBP competence. The specific research questions included:

- What is the mean score of the EBP-COQ scale, including subscales (EBP knowledge, skills, and attitudes) among nursing students?
- How does knowledge and skills in EBP and positive attitudes towards EBP affect students’ overall EBP competence?
- How do other sample characteristics including age, gender, academic year of study, and training related to research methodology affect overall students’ EBP competence?

It is also worth mentioning that there are no previous studies on a sample of students in Greece, as the competence has only been studied in registered nurses so far. However, the fact that only about 20% of Greek universities have integrated EBP courses into their curricula ranks Greece at a very low level of competence of undergraduate nursing students regarding EBP [20,23,24].

## 2. Materials and Methods

### 2.1. Study Design and Sample

This descriptive–analytical study was based on cross-sectional analysis which examined the level evidence-based practice competence and its relationship with demographic variables. A convenient sampling method was used to recruit the participants. The data were collected from February to June 2022 among a sample of undergraduate nursing students at the Hellenic Mediterranean University (HMU) in Greece. Students of the 2nd and 3rd academic years comprised the sample of this study because the lessons of EBP and research methodology are taught in these academic years. Also, the clinical trials that the students attend begin after the 3rd academic year.

### 2.2. Data Collection Tools

The data collection instrument was a two-section standard questionnaire. The first section of the questionnaire encompassed demographic information (including the gender, age, academic year, and training in the field of research methodology), and the second section contained the standard Greek version of the Evidence-Based Practice Competence Questionnaire (EBP-COQ), translated and validated into Greek from Spanish [25,26]. It is worth mentioning that the EBP-COQ tool, which was used in its Greek version in our study, has been weighted and translated in many countries of the world. Also, the translation of the EBP-COQ into Greek is of great importance as the use of the tool will significantly help to assess the competence of nursing students in the field of EBP and consequently will contribute to the improvement of the students’ level in terms of EBP. EBP-COQ was specifically developed to evaluate the self-perceived level of evidence-based practice competence among nursing students. The questionnaire consists of 25 items, which are organized in a three-factor structure. Factor 1: Attitude toward EBP (13 items), Factor 2: Skills in EBP (6 items), and Factor 3: Knowledge in EBP (6 items). Ιn the Greek version, Cronbach’s alpha was 0.81 for all items, 0.86 for Attitude towards EBP, 0.80 for Knowledge-Skills in EBP, and 0.78 for EBP Perceptions [25]. All items of the instrument are scored on a Likert-type scale of 1–5. The score of each subscale is calculated as the average of the corresponding items with a higher score indicating more self-perceived competence in EBP, greater self-perception of knowledge and skills in EBP, and more positive attitudes towards EBP.

### 2.3. Ethical Consideration

The present study was approved and examined by the Hellenic Mediterranean University Ethics Committee (no. 29/18.01.21). Moreover, the present study was conducted in accordance with the new General Data Protection Regulation (GDPR) (EU 2016/679) on sensitive personal data. The required licenses were obtained by the respective services prior to installation. The data obtained were anonymous, and their usage was limited to the survey and the principal researcher’s access to them. The participants gave their written agreement after being properly informed that the procedure was anonymous, that their personal data and replies would be used solely for research reasons, and that they may leave at any given moment. In addition, the permission to use the questionnaire was obtained from the original author of the Greek version of the instrument.

### 2.4. Data Analysis

The statistical analysis was performed using SPSS version 26.0 (SPSS Inc., Chicago, IL, USA). Continuous variables were expressed as mean ± standard deviation and categorical variables were expressed as numbers (percentages). An independent sample *t*-test was used to determine the difference in the mean score of the EBP-COQ scale and subscales regarding gender and academic year. The ANOVA test was used to investigate the difference between the mean scores of the EBP-COQ scale and subscales regarding their age category and level of training in the field of research methodology. To investigate the correlation between EBP-COQ subscales, Pearson’s correlation coefficient was used. Normality of the variables was tested by the Shapiro–Wilk test. For all tests, statistical differences were determined to be significant at *p* < 0.05.

## 3. Results

In total, 175 questionnaires were distributed (58 students of the 2nd and 117 undergraduate students of the 3rd academic year), of which 175 completed questionnaires were returned (a response rate of 100%). Most of the participants (57.33%) were in the 21–22-year-old age group (67.43%) and in the 3rd academic year (66.86%). Moreover, 82.29% of the participants were females, and the rest males. Most of the respondents had <40 h training in the field of research methodology. Table 1 shows the frequency distribution of the participants’ information.

As presented in Table 2, the mean score of EBP-COQ for nursing students was 3.03 ± 0.26, indicating moderate competence in EBP. Among the EBP-COQ dimensions, the mean scores were 3.03 ± 0.32 for Attitude toward EBP, 3.01 ± 0.49 for Skills in EBP, and 3.03 ± 0.49 for Knowledge in EBP.

The mean total EBP-COQ score in women (3.02 ± 0.26) did not differ significantly from that of men (3.04 ± 0.26), *p* = 0.766. Also, no significant difference was found between the age groups 18–20 (2.95 ± 0.21), 21–22 (3.04 ± 0.27), 23–24 (3.02 ± 0.27), and >24 (3.03 ± 0.23), *p* = 0.400. Regarding the academic year, no significant difference was found between second-year (2.99 ± 0.24) and third-year students (3.04 ± 0.26), *p* = 0.153. Finally, based on training in research methodology, no significant difference was found between students with no training at all (2.97 ± 0.28), those with <40 h of training (3.03 ± 0.25), and those with 40–150 h of training (3.07 ± 0.32), *p* = 0.538 (Table 3).

The average total score of EBP-COQ-Attitude among women (3.03 ± 0.32) did not show a statistically significant difference compared to men (3.07 ± 0.33), with a *p*-value of 0.451. Similarly, there was no significant variance observed among different age groups: 18–20 (2.96 ± 0.36), 21–22 (3.04 ± 0.32), 23–24 (3.06 ± 0.30), and >24 (3.11 ± 0.30), with a *p*-value of 0.464. In terms of academic year, no significant difference was detected between second-year (2.99 ± 0.32) and third-year students (3.05 ± 0.32), with a *p*-value of 0.258. Lastly, when considering training in research methodology, no significant difference was found between students without any training (3.14 ± 0.28), those with less than 40 h of training (3.02 ± 0.33), and those with 40–150 h of training (3.07 ± 0.32), with a *p*-value of 0.297. In addition, the mean total score of EBP-COQ-Skills in females (3.01 ± 0.50) did not show a statistically significant difference compared to males (3.01 ± 0.45), with a *p*-value of 0.992. Similarly, there was no significant variance observed among different age groups: 18–20 (2.99 ± 0.35), 21–22 (3.02 ± 0.50), 23–24 (2.87 ± 0.54), and >24 (3.16 ± 0.57), with a *p*-value of 0.494. In terms of academic year, no significant difference was detected between second-year (2.99 ± 0.46) and third-year students (3.03 ± 0.50), with a *p*-value of 0.623. Lastly, concerning training in research methodology, no significant difference was found between students without any training (2.88 ± 0.45), those with less than 40 h of training (3.01 ± 0.48), and those with 40–150 h of training (3.15 ± 0.54), with a *p*-value of 0.246. Lastly, the average total score of EBP-COQ-Knowledge in women (3.03 ± 0.50) does not show a significant difference from that of men (3.03 ± 0.44), *p* = 0.984. Additionally, there was no significant difference found between the age groups 18–20 (2.92 ± 0.44), 21–22 (3.07 ± 0.50), 23–24 (3.12 ± 0.50), and >24 (2.83 ± 0.51), *p* = 0.163. In terms of academic year, no significant difference was found between second-year (2.97 ± 0.43) and third-year students (3.05 ± 0.52), *p* = 0.314. Lastly, based on training in research methodology, no significant difference was found between students with no training at all (2.89 ± 0.46), those with <40 h of training (3.05 ± 0.48), and those with 40–150 h of training (2.98 ± 0.59), *p* = 0.393.

All aforementioned results are clearly depicted in Table 4.

The findings did not reveal a significant correlation between EBP-COQ subscales (Table 5). Specifically, no strong correlation was found between the attitude subscale and the skills (*p* = 0.483) and knowledge (*p* = 0.487) subscales, nor between the skills and knowledge subscales (*p* = 0.219).

## 4. Discussion

Ruzafa et al., who was the creator of the questionnaire, reports that the Cronbach’s alpha index for the entire questionnaire was 0.888 (0.940 for Attitude toward EBP, 0.756 for Skills in EBP, and 0.800 for Knowledge in EBP). Patelarou et al. also reports that the Cronbach’s alpha index is calculated at 0.811 for all items (0.858 for Attitude toward EBP, 0.789 for Skills in EBP, and 0.777 for EBP Perceptions). The value of using a translated form of the EBP-COQ in Greek is that it will significantly aid in the reliable interpretation of nursing students’ competence in EBP and, at the same time, provide incentives to improve their level in this regard [25,26]. Similar results have been found by other validation studies of EBP-COQ, such as the study of nursing and midwifery of Kermanshah and Ilam University of Medical Sciences in Iran, where Cronbach’s alpha was 0.7. In the study by Wang et al. where the EBP-COQ was weighted and translated into English, Cronbach’s alpha for the entire instrument was 0.83 [27,28]. In the study by Finotto et al. for the weighting of the EBP-COQ in Italian, Cronbach’s alpha has a value of 0.892 [29]. Additionally, in countries such as Turkey where the EBP-COQ was validated and Yildiz et al. created the Turkish version, Cronbach’s alpha was found to be 0.826 (0.850 for Attitude toward EBP, 0.516 for Skills in EBP, and 0.587 for Knowledge in EBP) [30]. A close value to Cronbach’s alpha was also found for the validation of the EBP-COQ which was carried out on nursing students in Colombia, at 0.89, while a similar value was also found in a study carried out in Poland, and the value of Cronbach’s alpha in this case was 0.856 for the entire questionnaire [31,32]. Therefore, based on all the above we can say that the tool we used in this research has a high degree of reliability, which is very positive for our study.

The present study highlighted important data on the competency of nursing undergraduate students regarding EBP in a Greek university. As mentioned in the three main aspects (attitude towards EBP, skills, and knowledge) that describe the level of undergraduate nursing students regarding EBP, the level of undergraduate students is characterized as average and this means a lot regarding their preparedness in the field of EBP. In more detail, the main findings of the present study regarding the competence of the undergraduate nursing students included mean scores of 3.03 ± 0.32 for Attitude toward EBP, 3.01 ± 0.49 for Skills in EBP, and 3.03 ± 0.49 for Knowledge in EBP.

Similar research findings exist in the bibliography and in other studies. For instance, the knowledge of Oman undergraduate university nursing students regarding the EBP had a value of 3.41 (SD = 0.66), their skills a value of 3.62 (SD = 0.51), and their attitudes a value of 3.41 (SD = 0.68) [33]. Additionally, the results from another study that was conducted on nursing students at a Spanish university seem to be more encouraging regarding their increased knowledge value, which is 4.23 (SD = 0.35), their skills at 3.93 (SD = 0.36), and their attitude at 4.34 (SD = 0.29) [34]. However, in an equivalent study there was a slight increase in the values of attitude, being 3.33, while the values of the skills (2.75) and knowledge (2.82) were lower than the findings of the present study [5]. Similar findings are reported in other studies, where undergraduate nursing students do not have a high level in these three aspects. For this reason, the need for proper guidance and encouragement from teachers to undergraduate students regarding the subject of EBP is highlighted [35]. In other studies, we find that there is a connection between the reduced competence of undergraduate students and the inadequate provision of patient care. So, the level of nursing care is judged according to the competence of the undergraduate students providing EBP [36,37,38].

At the same time, the lack of skills, especially found in bibliography searches and critical evaluation of data, reduces undergraduate students’ competence percentage regarding EBP, regardless of whether they themselves have a high percentage of understanding regarding the necessity of applying EBP in nursing and confidence in the subject of EBP [38,39]. Moreover, it is mentioned that even undergraduate students who have some knowledge about EBP consider that they lack that knowledge and time and have a negative attitude [40]. Due to the reduced competence of undergraduate students towards EBP, it is of utter importance to educate undergraduate nursing students regarding the strengthening of critical thinking, proper bibliography search techniques, and evaluation of research data [11,41,42]. Having EBP courses in the academic curriculum will improve undergraduate students’ EBP competence in knowledge, attitude, and skills towards EBP [5]. It would be very important to improve the EBP learning techniques and methods in the future. One such example is the effort to implement the EBP e-Toolkit in EBP courses for the education of undergraduate nursing students in Greece.

It is true that the curricula related to the teaching of EBP at the undergraduate level differ between universities, both in Greece and in other countries [20,25]. A related study was carried out among six European countries (Czech Republic, Greece, Italy, Poland, Slovenia, and Spain) and a total of 162 higher education nursing departments. Regarding the teaching of EBP in undergraduate curricula, only about 30% of these nursing departments appear to be responsive in their curricula. EBP is one of the newest disciplines in nursing and in higher education curricula, which explains the lack of a high rate of integration into them, as well as the lack of common methods and guidelines regarding EBP teaching methods in the study programs. It is characteristic, that in Greece, where our own study was also carried out, of the ten higher education departments of nursing that exist, only two of them include courses related to EBP in their curriculum, in contrast to, for example, the Czech Republic which maintains a fairly high ratio [20].

It is evident from this study and from other ones that are related to the competency of undergraduate students in the EBP field that there is a need for change. That requires a lot of effort for nursing universities to overcome emerging barriers and increasingly integrate EBP into the curricula as the needs for evidence-based clinical patient care will continue to grow. At this point it is worth mentioning that the relatively modest level of the undergraduate nursing students in EBP competence is largely due to the strong attachment that dominates the educational and clinical field regarding the anachronistic and traditional concepts of applying nursing practices [43,44,45]. This fact significantly discourages undergraduate nursing students from adopting and implementing EBP and, as a result, clinical practices are not based on indicators with any negative implications this entails. Many studies point out that addressing this issue needs strategy, i.e., with dynamic interventions in the field of EBP in the education programs of undergraduate nursing students. An example of such an intervention could be the creation of a separate course in the study cycle exclusively for EBP. This will be an important solution and will greatly improve the competency levels of undergraduate nursing students, as they will acquire specialized knowledge and skills in the field of EBP and, therefore, they will have a greater performance later on in making and implementing their clinical decisions [43,44,45,46,47].

At this point, reference should be made about the limitations of the present study, as it is not multicentered. The research was conducted at a single university, which limits the generalization of the results to other institutions and educational contexts in Greece. For this reason, there were limitations regarding this type of research as mentioned above, as there is currently no other such study regarding the teaching of EBP, so there were no data for analyzing and comparing corresponding studies involving Greek university students. At the same time, however, this is a motivation to carry out similar studies in Greece, where the present study was conducted in just one nursing university. It would be important after we carry out the assessment of the effectiveness of the intervention to extend the research to a larger sample in order to obtain a better picture on this topic. Also, the fact that in the present study there was an absence of statistically significant differences makes us think that possibly more teaching hours are required for the EPB course in order to achieve a positive correlation.

## 5. Conclusions

The present research was carried out in order to evaluate the readiness of undergraduate nursing students at a Greek university. A sample of undergraduate nursing students was used and, with the help of the psychometric tool EBP-COQ, important data were obtained about the attitude, knowledge, and skills of the undergraduate students regarding EBP. From the resulting research data, it appears that the level of readiness towards EBP is mediocre. Therefore, it is necessary to carry out new studies in the future, so that there is a correct approach of all the elements that contribute to the readiness of each undergraduate nursing student regarding EBP, because the greater the readiness in EBP care is, the better the quality of patient care. This particular study and similar ones in the future will help researchers to assess and evaluate the level of competence of nursing students regarding EBP in order to organize dynamic interventions and be more effective in improving the level of competence of nursing students in the field of EBP. Such interventions may concern the teaching methods of EBP courses, the media used for teaching, the total teaching hours, etc. so that there is a better level of competence in EBP.

## Figures and Tables

**Table 1:** Frequency distribution of participants’ information (*n* = 300).

		Count	Column N %
Biological sex	Female	144	82.29%
	Male	31	17.71%
Age	18–20	31	17.71%
	21–22	118	67.43%
	23–24	12	6.86%
	>24	14	8.00%
Academic year	2nd	58	33.14%
	3rd	117	66.86%
Training in the field of research methodology	None	17	9.71%
	<40 h	138	78.86%
	40–150 h	20	11.43%

**Table 2:** Mean and standard deviation of participants’ EBP-COQ scores.

	Score Domain	Mean	Standard Deviation
EBP-COQ	1–5	3.03	0.26
Attitude toward EBP	1–5	3.03	0.32
Skills in EBP	1–5	3.01	0.49
Knowledge in EBP	1–5	3.03	0.49

**Table 3:** Relationship between EBP-COQ regarding participants’ information.

		EBP-COQ	
		Mean ± Standard Deviation	Test, *p*-Value
Gender	Female	3.02 ± 0.26	t(173) = −0.298, *p* = 0.766
	Male	3.04 ± 0.26	
Age	18–20	2.95 ± 0.21	F (3, 171) = 0.987, *p* = 0.400
	21–22	3.04 ± 0.27	
	23–24	3.02 ± 0.27	
	>24	3.03 ± 0.23	
Academic year	2nd	2.99 ± 0.24	t(173) = −1.436, *p* = 0.153
	3rd	3.04 ± 0.26	
Training in the field of research methodology	None	2.97 ± 0.28	F (2, 172) = 0.622, *p* = 0.538
	<40 h	3.03 ± 0.25	
	40–150 h	3.07 ± 0.32	

**Table 4:** Relationship between EBP subscales regarding participants’ information.

		Attitude toward EBP		Skills in EBP		Knowledge in EBP	
		Mean ± Standard Deviation	Test, *p*-Value	Mean ± Standard Deviation	Test, *p*-Value	Mean ± Standard Deviation	Test, *p*-Value
Gender	Female	3.03 ± 0.32	t(173) = −0.755, *p* = 0.451	3.01 ± 0.50	t(173) = 0.010, *p* = 0.992	3.03 ± 0.50	t(173) = 0.020, *p* = 0.984
	Male	3.07 ± 0.33		3.01 ± 0.45		3.03 ± 0.44	
Age	18–20	2.96 ± 0.36	F (3, 171) = 0.858, *p* = 0.464	2.99 ± 0.35	F (3, 171) = 0.803, *p* = 0.494	2.92 ± 0.44	F (3, 171) = 1.728, *p* = 0.163
	21–22	3.04 ± 0.32		3.02 ± 0.50		3.07 ± 0.50	
	23–24	3.06 ± 0.30		2.87 ± 0.54		3.12 ± 0.50	
	>24	3.11 ± 0.30		3.16 ± 0.57		2.83 ± 0.51	
Academic year	2nd	2.99 ± 0.32	t(173) = −1.136, *p* = 0.258	2.99 ± 0.46	t(173) = −0.493, *p* = 0.623	2.97 ± 0.43	t(173) = −1.009, *p* = 0.314
	3rd	3.05 ± 0.32		3.03 ± 0.50		3.05 ± 0.52	
Training in the field of research methodology	None	3.14 ± 0.28	F (2, 172) = 1.223, *p* = 0.297	2.88 ± 0.45	F (2, 172) = 1.415, *p* = 0.246	2.89 ± 0.46	F (2, 172) = 0.938, *p* = 0.393
	<40 h	3.02 ± 0.33		3.01 ± 0.48		3.05 ± 0.48	
	40–150 h	3.07 ± 0.32		3.15 ± 0.54		2.98 ± 0.59	

**Table 5:** Correlation between EBP-COQ subscales.

		Attitude toward EBP	Skills in EBP	Knowledge in EBP
Attitude toward EBP	Pearson Correlation	1	−0.053	−0.053
	Sig. (2-tailed)		0.483	0.487
	N	175	175	175
Skills in EBP	Pearson Correlation	−0.053	1	0.093
	Sig. (2-tailed)	0.483		0.219
	N	175	175	175
Knowledge in EBP	Pearson Correlation	−0.053	0.093	1
	Sig. (2-tailed)	0.487	0.219	
	N	175	175	175

## Data Availability

The data presented in this study are available on request from the corresponding author.
